# Women’s freedom of movement and participation in psychosocial support groups: qualitative study in northern India

**DOI:** 10.1186/s12889-019-7019-3

**Published:** 2019-06-10

**Authors:** Nicola Gailits, Kaaren Mathias, Elysée Nouvet, Pooja Pillai, Lisa Schwartz

**Affiliations:** 1 Dalla Lana School of Public Health, University of Toronto, 155 College St., Toronto, Ontario M5T 1P8 Canada; 2 Emmanuel Hospital Association, 808/92 Deepali Building, Nehru Place, Delhi, New Delhi 110019 India; 3 School of Health Studies, Western University, Labatt Health Sciences Bldg, Rm 215. 1151 Richmond St., London, ON N6A 5B9 Canada; 4 Department of Health Research Methods, Evidence and Impact, McMaster University, 1280 Main Street West, Hamilton, Ontario L8S 4K1 Canada

**Keywords:** India, Depression, Women’s health, Qualitative Research, Community Mental health, Gender relations, Global Mental health, Psychosocial factors, Women’s autonomy

## Abstract

**Background:**

Depression, the world’s leading cause of disability, disproportionately affects women. Women in India, one of the most gender unequal countries worldwide, face systemic gender disadvantage that significantly increases the risk of common mental disorders. This study’s objective was to examine the factors influencing women’s participation in psychosocial support groups, within an approach where community members work together to collectively strengthen their community’s mental health.

**Methods:**

This community-based qualitative study was conducted from May to July 2016, across three peri-urban sites in Dehradun district, Uttarakhand, Northern India. Set within an NGO-run mental health project, data were collected through focus group discussions with individuals involved in psychosocial support groups including women with psychosocial disabilities as well as caregivers (*N* = 10, representing 59 women), and key informant interviews (*N* = 8) with community members and mental health professionals. Data were analyzed using a thematic analysis approach.

**Results:**

The principal barrier to participating in psychosocial support groups was restrictions on women’s freedom of movement. Women in the community are not normally permitted to leave home, unless going to market or work, making it difficult for women to leave their home to participate in the groups. The restrictions emanated from the overall community’s attitude toward gender relations, the women’s own internalized gender expectations, and most significantly, the decision-making power of husbands and mothers-in-law. Other factors including employment and education shaped women’s ability to participate in psychosocial support groups; however, the role of these additional factors must be understood in connection to a gender order limiting women’s freedom of movement.

**Conclusions:**

Mental health access and gender inequality are inseparable in the context of Northern India, and women’s mental health cannot be addressed without first addressing underlying gender relations. Community-based mental health programs are an effective tool and can be used to strengthen communities collectively; however, attention towards the gender constraints that restrict women’s freedom of movement and their ability to access care is required. To our knowledge, this is the first study to clearly document and analyze the connection between access to community mental health services in South Asia and women’s freedom of movement.

**Electronic supplementary material:**

The online version of this article (10.1186/s12889-019-7019-3) contains supplementary material, which is available to authorized users.

## Background

### Indian women’s mental health, gender inequality, and access to care

Major depression is the leading cause of disability worldwide [1], with mental, neurological and substance use disorders accounting for 10.4% of global DALYs [2]. Within India, a meta-analysis revealed that mental and behavioural disorders have a prevalence of 8.9% [3], while the prevalence of depression in a study in urban South India was 15.1% [4]. For women, the worldwide burden of depression is 50% higher than for men [5].

India is one of the least gender equal countries in the world: the Global Gender Gap report placed India in 87th place worldwide, and even lower in economic participation and opportunity (136th) and health and survival (142nd), out of 144 countries [6]. For Indian women, there are culturally specific and socio-economic causes for depression that must be examined in the context of gender inequality. The rigid and traditional roles women have in Indian culture restrict their agency and lower their social status [7]. A study examining gender and health disparities in South Asia emphasized how South Asian women are often excluded from decision-making and have little control over resources [8]. The study’s authors state that Indian women are often undervalued because they are not seen as “making a visible economic contribution” to their household [8]. Gender inequality in India impacts both women’s experience of mental health and options to access health care. Women are less likely to seek out proper care for diseases [8], even though, as in the case of depression, they are disproportionately affected and many experience the “double burden of gender disadvantage and poverty” [9]. In response to high levels of distress, interventions focused on the provision of social support for Indian women have been shown to improve their mental health [7, 10].

While depression is a leading cause of disease burden in India [11], 90% of the Indian population with mental health problems cannot access evidence-based mental health services [12]. In the Northern state of Uttarakhand, a cross-sectional study of individuals with depression demonstrated a complete lack of access to talk therapy, and only 3% of people with depression had accessed anti-depressants [13]. This context of a high treatment gap and few mental health resources suggests the importance of alternative responses: community-based mental health care can provide an effective response in low resources settings, as it is less costly, does not require mental health professionals and can be contextually acceptable to the community [14, 15]. Recent task-shifting models have demonstrated the effectiveness of using non-specialized workers in the provision of mental health care [15]. In light of the need to support community mental health (CMH) models, this study was framed within a CMH theoretical framework.

### Theoretical framework: CMH competence

Campbell and Burgess [16] advocate for the importance of building *CMH Competence*, defined as: “the ability of community members to work collectively to facilitate more effective prevention, care, treatment and advocacy” for mental health. Under their definition, being able to cope in difficult conditions requires access to political, economic, or psychological resources [17]. In low resource settings, these resources may not be available and therefore coping with life stresses becomes increasingly difficult, and eventually impacts health status. Their community mental health competency framework highlights the ability of communities themselves to increase health-promoting behaviours and decrease stress [17]. This framework includes three core dimensions: knowledge, safe social spaces, and partnerships for action [18]. Knowledge includes recognizing symptoms and accessing services, while safe social spaces focus on discussion and social inclusion in the community. Partnerships and collective action examines relationship formation, health-promoting behaviours, and collective action for mental health [19].

### Psychosocial support group (PSSG) interventions

One application of CMH Competence is community-based psychosocial support groups (PSSGs) as one way to increase social support and reduce depressive symptoms [20–24], while building on community resources for care. The majority of studies providing PSSG or PSSG-like interventions primarily worked with populations living with HIV, mostly in the African context [20–23, 25]. In the this context, PSSGs have provided women with emotional assistance and coping [26], and improved mental and physical health, including reducing depression and increasing functionality [20–22].

However, very few studies have examined PSSGs in the Indian context. One study with injecting drug use widows in Eastern India used participatory action groups to promote mental health and reduce risky HIV behaviours [23]. After 10 sessions, the proportion of women experiencing a common mental disorder decreased from 70 to 42% [23]. Another study, conducted in Southern India, combined a mental health intervention with a microcredit economic activity and found a reduction of psychological symptoms and increases in social support [27]. In Pakistan, a South Asian country where women have similarly low levels of autonomy [28], two randomized control trials found PSSGs to be beneficial: one 6-week social support intervention found women improved their mental health and resilience [29], and one 5-week Group PM+ intervention found significant improvements in anxiety, depression, and psychological wellbeing [30].

While these studies advance understanding on the potential impact of PSSGs for South Asian women’s mental health, only one study included any mention of participation barriers. The Group PM+ intervention found three barriers to participation: session time length (2 hours was too long to be away from home), monetary compensation (no compensation meant less motivation to attend), and confidentiality (having two family members from same household limited participation) [30]. Besides this mention, no study has examined factors that influence participation in PSSGs in the South Asian context, and this represents a clear gap in the literature.

Understanding the factors that influence women’s participation in PSSGs is important for two reasons. Given the high mental health treatment gap with minimal access to mental health services in Northern India, for a community-based service such as PSSGs that does exist, it is important to understand how to enable more women and communities to participate fully. This becomes even more crucial in the context of high levels of gender inequality that inhibit women’s access to healthcare.

### Study objective

This study was partnered with the non-governmental organization (NGO) Emmanuel Hospital Association and their mental health project, Burans. In 2014, Burans began running women’s psychosocial support groups (PSSGs), alongside other community interventions including community awareness building, and individual counseling. This study was part of a larger research project examining successes and challenges of building CMH competence in Dehradun district, Uttarakhand (Northern India).

This study’s initial objective was to examine the successes and challenges of women’s PSSGs, and ways PSSGs worked to build CMH competence. Women participants in PSSGs included those with personal experience of mental health problems as well as female caregivers who, in this context, experience psychological and social distress that is not experienced by male caregivers [31].

However, during the research process, it became apparent that there were significant challenges for women in the community to access and participate in the support groups in the first place. As such, the study’s objective shifted towards examining the complex factors influencing North Indian women’s participation in PSSGs, all within an overall objective of building CMH competence.

As stated above, no previous studies have examined women’s motivations and barriers to participating in psychosocial support groups in South Asia. Outside of this specific intervention, it is important to note that there is a general paucity of mental health research from Northern India. The majority of Indian mental health research emerges from Southern states, with uncertain transferability to Northern India [32]. This research becomes even more important when considering that women in Northern India are more disadvantaged than those from Southern regions; they experience more restrictions on their autonomy and freedom of movement, and have fewer inheritance rights [28].

## Methods

This community-based qualitative study aimed to build on the community’s strengths and resources and generate research relevant to community members. Key methods of data collection included focus group discussions (FGDs) and key informant interviews (KIIs).

### Setting and intervention

PSSGs involve hour-long meetings of 5–10 women every 2 weeks. Their aim is to collectively learn about mental health concepts and self-care as well as to provide a safe space to share experiences, strengthen relationships and networks in the community, and create opportunities for social support. Groups were formed by Burans’ community health workers (CHWs) trained in mental health knowledge, group facilitation, and counselling skills. CHWs invited women in neighbouring communities to join the group. They facilitated a nine-module curriculum in the form of a flipchart, covering topics including positive thinking, tension, resilience, depression, sleep, and how to take action on your mental health for yourself and your community. There were separate groups for people with mental and psychosocial disability (PPSD), and women who are caregivers of PPSD. PPSD is the preferred term in the community, and can be defined as “people who have received a mental health diagnosis, and who have experienced negative social factors including stigma and discrimination and exclusion” [33]. PPSD support group participants were limited to those with common mental disorders.

Burans runs PSSGs across three diverse sites in the Dehradun district of Uttarakhand: Mussoorie, Dehradun, and Sahaspur. Mussoorie is located in a mountainous region, where women were predominantly Hindu. In urban Dehradun, women were more often employed, some working seasonally for 6 months of the year as brick makers, and were a mix of Hindu and Muslim faith traditions. The third site, Sahaspur, was a peri-urban village area located on the plains outside of Dehradun, and women were predominantly Muslim and unemployed. Table 1 provides an overview of the demographics in the study area, and shows several clear indicators of structural gender disparity in the sex ratio at birth and difference in literacy rates between men and women.

**Table 1:** Socio-Demographic Profile of the Study District

Indicator	National - India	State-Uttarakhand	District-Dehradun
Population (millions)	1210	10.1	1.7
Rural Population	68.8%	69.5%	44.5%
Under Age 15 Population	28.6%	28.9%	25.3%
Sex Ratio at Birth (females: males)	919: 1000	888: 1000	886: 1000
Female Literacy Rate	68.4%	76.5%	81.5%
Male Literacy Rate	85.7%	90.7%	91.8%

State and National level data from 2015 to 16, Dehradun district data from 2011 to 13. Sources: [34–37]

### Sampling and inclusion

Given that the study was examining an intervention, total population sampling was used. Using this particular type of purposeful sampling means that the sample size included all psychosocial support groups run by Burans. The majority of these groups had been active at least 6 months and could therefore better reflect on the impact of participating in the support group. Within PSSGs, individual inclusion criteria included: Hindi-speaking women impacted by common mental disorders either personally or as a caregiver of a family member, and capable of giving consent. A mix of caregiver and PPSD groups were included. Additionally, KIIs were conducted to get a better understanding of the local mental health context. KIIs included Burans staff members, CHWs, a psychiatrist, a mental health nurse, a counsellor, and a PSSG group leader. KIIs included adults over 18, who spoke either Hindi or English.

### Data collection

In total, ten FGDs were conducted over two rounds with seven unique PSSGs, representing 59 women. In developing questions to guide the FGDs, the order, wording, and potential cultural sensitivity of certain topics was taken into account (see full guides in Additional files 1 and 2). Most importantly, the guides were developed in dialogue with local partners, in order to ensure that questions posed were culturally appropriate and suitable for limited literacy adults. The initial FGD guide explored 10 questions related to PSSG impact, success factors, collective mental health action as a result of PSSGs, future PSSG formation improvements, as well as long term sustainability of groups. A first round of FGD questions were chosen based on the CMH Competence framework, and the need to examine the successfulness of the PSSGs in order to strengthen future support groups.

Even though we came into the study ready to explore the successfulness and future sustainability of PSSGs in the community, after analyzing data from the first round of FGDs, it became apparent that many women in the community had significant difficulties being able to leave their home to attend the support group. It was at this point that the study’s focus shifted to a more important research question: “why were some women unable to participate in the PSSGs, and what other factors were enabling or preventing their participation?” As such, we conducted a second round of FGDs in order to deepen understanding on this central theme: women’s freedom of movement.

Participation in FGDs was voluntary. After gathering the women together, PP explained that the purpose of the study was to understand any positive or negative impacts of participating in the PSSGs, with an overarching objective of learning from participants. As individuals experiencing the program first hand, this could also be seen as an opportunity to benefit from program improvements or support sustainability. After the study was explained and any questions were answered to the entire group, each woman in attendance was provided with the opportunity to orally consent or not, to participation in the study. Since women were not normally paid to attend PSSGs, monetary compensation was deemed inappropriate for participants by the director of Burans. However, food and beverages were provided as curtesy for their time. FGDs were audio-recorded, and lasted from 45 to 70 min. PP facilitated the FGDs in Hindi while NG noted the group’s atmosphere, and non-verbal responses. After each FGD, NG and PP recorded a debrief session to brainstorm major themes and improve the research process. The recordings were later written into memos, significantly aiding the analysis process. After the first round of seven FGDs, NG and PP returned to three of the support groups for a second round of FGDs, in order to gain further information specific to the main preliminary finding on women’s inability to leave the home.

Eight KIIs were also conducted, some in English and some in Hindi, to provide further context on community mental health awareness and gender roles (see Additional file 3 for KII guide). Participants were asked questions about women’s independence in the community context, PSSGs’ ability to initiate collective action, successful PSSG formation process, and PSSG sustainability.

### Data analysis

Data was translated and transcribed from Hindi to English, checked for accuracy, and anonymized. Thematic analysis as outlined by Braun and Clarke [38] was used with the support of QSR Nvivo 11 software. After the first round of FGDs, there was a preliminary analysis stage where PP and NG separately coded four transcripts (mix of KII and FGD), developing preliminary codebooks. These codebooks were compared, and consensus was reached on differences for the naming and inclusion of key emerging themes, with a third analyst familiar with the local setting, KM, providing additional insight for this stage of the analysis.

After round two of FGDs, an initial concept map emerged, outlining the key themes and their interconnectivity. NG then coded the rest of the data, ensuring each transcript was read at least twice. All data was coded, and themes were found to be consistent across both KIIs and FGDs. Once coding was complete NG, LS and EN further analysed the data’s themes, and developed the concept map.

Both inductive and deductive coding were used with the most pertinent codes emerging inductively. Nvivo 11’s memos were used significantly: for each FGD or KII, one memo provided context for the data from the transcribed debrief that had occurred directly after each FGD. A second memo was written as data was coded, to brainstorm and describe main themes emerging from the data.

## Results

**Fig. 1:**
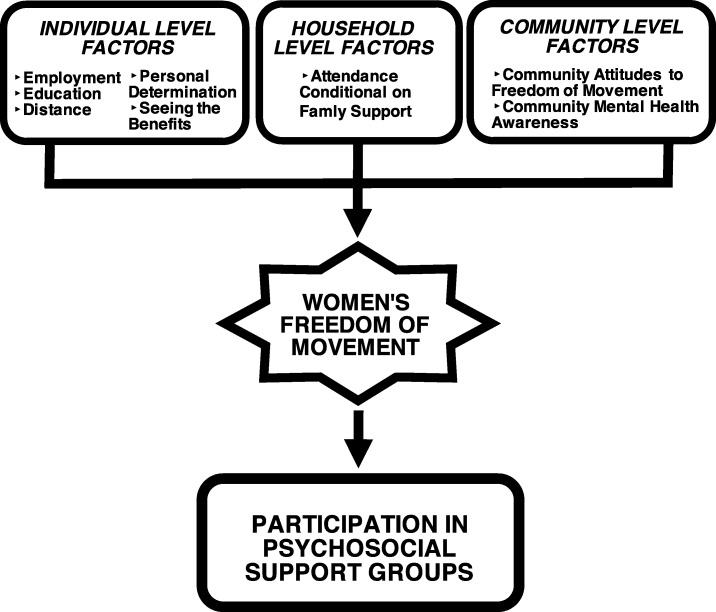
Factors Influencing Psychosocial Support Group Participation

We identified several factors that impacted women’s participation in psychosocial support groups. These factors emerged as different yet inextricably connected spheres in women’s lives: at the community, household, and individual levels. All factors indicated the significance of uneven gender relations, and related to a main factor: freedom of movement, or women’s autonomy to leave the home in order to attend PSSGs. Therefore, women’s freedom of movement emerged as the central gatekeeper for women’s participation in PSSGs (see Fig. 1). In what follows, we describe each participation factor through the lens of this primary influence.

Examination of the demographics of women participating in PSSGs showed that older women (40–60 years old) were more often uneducated, and the younger women (20–30 years old) had often completed primary or high school. Two-thirds of participants had been to school at varying levels, whereas one third had never been to school (see Table 2 for complete demographics).

**Table 2:** Demographics of Psychosocial Support Groups Involved in FGDs

FGD Location and Number*	Caregiver/PPSD	Months Since Group Start	Meetings/ Month	Members	Age Range	Average Age	Education Level			
							No School	Completed Primary	Completed Secondary	Completed Bachelors or Masters
DEH 1	Caregiver	2	2	11	18–45	31.0	2	3	6	0
DEH 2	Caregiver	8	2	13	19–65	34.4	3	1	3	6
MUS 1	Mixed	5–6	2–4	6	24–52	39.2	3	1	2	0
MUS 2	PPSD	8–9	2	5	26–49	34.8	1	3	1	0
MUS 3	PPSD	6–7	2	5	42–60	45.8	2	1	2	0
SAH 1	Mixed	7–8	1	9	25–65	35.8	4	4	0	1
SAH 2	Caregiver	5–6	2–3	10	16–36	23.4	2	6	2	0
Education Level Totals							17 No school	19 Primary	16 Secondary	7 Bachelor's/Masters
**Totals**				**59**	**16–65**	**33.6**	**29% No School**	**32% Primary**	**27% Secondary**	**12% Bachelors/Masters**

*DEH Dehradun, *MUS* Mussoorie, *SAH* Sahaspur

Totals are set in bold

### Community level factors

#### Community attitudes to freedom of movement

Both KII and FGD participants described traditional gender relations, for example, that a woman’s role is mainly to cook and remain at home while men are permitted to go out and return home when they desire. Generally, the hierarchy commonly accepted in the community is that men decide when and where women are allowed to go. Women stated that they are questioned if they leave home, as most participants did not work outside the house. When women joined the support group, some believed that they were going against the community’s role for women.

Participants described the male dominated society in India as a reason for the restriction of women’s freedom of movement. One male interviewed shared that often men in his community blame their own father or mother for the reason their wife cannot leave the house, when it is they themselves who feel their wives should not leave. For example, one woman’s son did not allow her to leave the house after her husband passed away because he thought people would be suspicious of what she was doing. More generally, according to participants, families do not like women to leave the home unless it is with a purpose they deem admissible. It is particularly difficult for a woman to leave the house for the first time because family members question why she is now suddenly leaving the home.

*Group Member (GM): “First we will have to seek permission from our husband, if he allows us to go.”*

*Community Health Worker (CHW): “People feel bad and do not like it. They say that women used to stay at home and now these people are taking her out (Logon ko accha nahi lagta hai. Pehle auratein ghar pe hi rehti thi. Ab yeh log unko ghar se bahar le jathe hai).” [Here, when the CHW says “these people are taking her out,” she is referring to the Burans staff members and their encouragement of women to participate in the community’s PSSGs, despite other community members’ disapproval.]*

In terms of the broader community, women participating in FGDs stated that some community members diminish women who join the support groups. They are suspicious of women leaving home, and question why they are getting together in groups. This makes it difficult for some women to participate in PSSGs. Below, one woman told stories of being followed when she was outside the home. Another described how families judge whether or not their daughter or wife should leave the house based on what their neighbours or other community members are doing.

*GM: “Even if we go to temple, from there they follow us. That’s why we feel scared in coming out. This is how it is in our community.”*

*Facilitator: “Who are the ones stopping women from coming out of the home?”*

*CHW: “In my opinion they are the father and mother-in-law. They are old and in that age, they think that if another person’s daughter-in-law doesn’t go, then how come our daughter in law can go out to purchase goods? They always compare with others in their life.”*

These restrictions on women’s movement have important impacts on the women’s lives, including their health, and the lack of ownership they feel over their health. One mental health professional interviewed explained that if a woman needs to go to the doctor for example, her husband must suggest it and would need to accompany her. Nevertheless, some women in the community persisted, attending the support groups and challenging these norms.

Additionally, participants highlighted the contribution of different religious traditions as a factor that influences women’s freedom of movement. All respondents agreed that it is more difficult for women from Muslim families to leave their homes in comparison to those in Hindu households, although both communities experienced restricted freedom of movement.

Overall, while the community’s norms for women’s freedom of movement are important, the actual restrictions or decisions were mediated through family members. However, women have also internalized the community’s gender norms: therefore, “not allowed to leave” has several meanings, in that some women described feeling bad for leaving the home. Violence was not mentioned, and while it seemed that women were rarely physically restrained from leaving the house, their perception was of an external force prescribing behaviour. The next section on household level factors discusses the direct role of specific family members in deciding whether women are permitted to leave the home.

#### Community mental health awareness

Before Burans came to the communities, many community members did not know that mental health or illness existed. Alongside PSSGs, Burans has been conducting awareness meetings with community members as well as religious and political leaders. Women explained that community members often refer to PPSDs as *paagal,* (mad or crazy); they told stories of being mocked in their community, and how some PPSDs are excluded from community events. Some women described how they have not told the community what they are doing in the support groups and that community members around them do not know what their groups are about.

Mental health stigma within the community may make it even more difficult for women to leave the house, as some participants believed that attending a PSSG might not be a valid reason for leaving the house. Even if the benefits of the group are explained, it was suggested that families may only want financial, not intellectual benefits to come from participation in a women’s group, since most families are lower income and need to prioritize household income opportunities. As a result, community members believe that the women are wasting time and not helping their household financially, as stated below:

*CHW: “They only think that it is something to 'pass the time' because they want some support for their family if the woman goes out of the house.” [Here, “support” refers to financial support]*

### Household level factors

#### Attendance conditional on family support

As stated above, participants indicated that the family held the ultimate decision-making power on whether a woman was permitted to go out or not, and therefore, whether or not she could participate in the PSSGs. The mothers-in-law or husbands were described as the primary decision maker. Those living in joint families with other elders found it more difficult to leave when elder women were present. Participants shared that for a woman to leave home, other family members may need to help with housework. Some families were unaware that women in their family were attending PSSGs. When asked how to help women attend PSSGs, the majority of participants emphasized that engaging the family is the most important approach.

In terms of restrictions to attend PSSGs, one group member commented:

*GM: “My own sister couldn’t come because her husband was not allowing her to.”*

Some women admitted to having trouble getting permission from family to leave the home, and even lying to their families in some cases. In order to attend, these group members made excuses of needing to buy something at the market and then they came to the meeting instead. It was apparent that resisting the community’s attitudes was difficult, however, some women saw the value in the PSSGs, and went to great lengths to attend.

*GM4/GM5 together: “Our family always has problems. They really wish that we would not go to the meetings.”*

*GM4: “They tell us not to go.”*

*GM5: “We like it, so we come.”*

### Individual level factors

An individual is part of a household, and every household exists within a community. The factors below shape the role individual women play in the household and the power she holds to make decisions. These decisions are validated or rejected by the community, based on the attitudes present. Therefore, these individual level factors often cannot be disentangled from the household and community factors. This section will begin with the most impactful participation factors within the individual level: employment and education.

#### Employment and income contribution

The possibility of leaving the house and participating in PSSGs was influenced significantly by whether a woman was employed, since for women who do work, their families were used to them going out. Some group members who were in paid employment said that they stayed out all day for work, and attended the support group at some point during the day. Therefore, for employed women, participation was much easier.

#### Education

Education was revealed to be an extremely important factor impacting participation, both in ability to attend the group and ability to contribute and participate. The more educated women in the groups tended to be younger women. Education and employment were intricately linked: similar to those employed, those with higher education levels participated more often and more confidently within the groups, were able to see the benefits of the groups easier, and had more ease in leaving the home to attend the group. One CHW said that initially, families never used to let women out but now more and more, and with increased education, their children are able to go out.

#### Seeing the benefits

Group members explained that it was only after experiencing the benefits of the PSSGs that they were able to see how important the support groups were and how they affected their mental health. Only then could they be able to advocate for more PSSGs in the community. This was one of the only themes that emerged that was not related directly to women’s freedom of movement. At the individual and family level, seeing the benefits was important to help enable and motivate women to attend the group. Participants stated that only once other women see the group’s benefits will they come out of their homes. They suggested that if women or program leaders explained the benefits to her own family, or other women and their families, it may help increase participation, as described below:

*CHW: “The main thing is when the woman comes to the group, she should know the truth about the support group as to what is the purpose of the group. The woman should know the benefits of the group, what she can achieve and where she can take herself through the support group. I believe that women can speak with their parent in laws once they understand all these things about the group.”*

#### Distance

Generally, women in the community do not travel far from home unaccompanied. According to participants, some women can travel to the market alone, and those who have jobs, may be able to travel greater distances. Distance appeared to be a factor determining whether women will be able to attend PSSGs. For this reason, PSSGs are hosted near women’s homes, normally in a member’s house who lives near others.

*Interviewer: "Are women allowed to go to Dehradun [a nearby city], or are their movements restricted to the village?*

*CHW: Many women do go to the hospital or the market. Some of them need someone to accompany them as they cannot go out on their own. Now that they are a part of the support group, they think they need to come out of their homes. The families' mentality is changing (parivar ki mansikta badal rahi hai), but it is difficult as knowledge is limited."*

#### Personal determination

Lastly, women’s determination to leave the home impacted participation. Group members interviewed stated that women must have a personal drive to leave the house. One CHW explained that for a woman who has never been outside her home, the biggest hurdle is making the first step to leave. Some group members interviewed spoke of their very strong self-determination, which they believe helped them to be able to leave the home to participate. Participants described the possibility for women to show agency and to resist the gender order that limited their movement, as described below:

*GM: “It depends on us. If we make a decision to do something, then not just family, even an outsider cannot say anything. First our spirits should be high (apne hausle buland hone chahiye). If we keep thinking ourselves that they will not allow us, they will interfere. But, if we said once that, we have to go, then we have to go. We have to strengthen ourselves first.”*

However, it is difficult to separate women’s determination to leave home from the community’s gender norms. Gender roles may be internalized to the point where women may not give themselves permission to leave, and determination may be influenced by tacit or conscious calculation of social, marital, or physical consequences should they act outside expected gender norms.

## Discussion

This research examined factors affecting women’s participation in psychosocial support groups in Northern India. Restrictions on women’s freedom of movement emerged as the most dominant barrier to PSSG participation. This barrier directly related to the community’s normative and unequal gender relations. The CMH Competence framework describes how communities in low resource settings can work together to achieve better prevention, care, and treatment for mental health [16] and underlines how a “powerlessness or a ‘lack of control over destiny’ severely undermines the health of people in chronically marginalised or demanding situations” [17]. This study demonstrates how building CMH Competence must start with a deeper understanding of power relations and how all members of the community, including women, can exercise autonomy to improve their mental health.

### Women’s freedom of movement

Restrictions on women’s freedom of movement is prevalent throughout India: India’s National Family Health Survey found that “only one-third of women age 15-49 are allowed to go alone to the market, to the health centre, and outside the community” [39], and another study found that 71% of Indian women have to ask for permission to leave the home [40]. Our study focused on the link between women’s mobility and access to health. While this has been confirmed by other studies in India [32, 39–41], few have been qualitative studies, and no other studies have looked at mobility and mental health. Other research in India has examined the link between gender disadvantage more broadly and rates of common mental disorders [9], as well as how gender impacts the determinants of mental health such as access to resources and social roles [42].

A recent systematic review in LMICs found that low levels of autonomy for women (including freedom of movement) were associated with poorer mental and physical health, and that this is possibly mediated through key determinants of health, including limited access to health services and education [43]. Our findings build on this idea, as it became clear that restrictions to women’s freedom of movement was a barrier to support group participation, and therefore limited women’s access to health and health education in the community. A maternal health care study in Northern India found that one of the most important factors for healthcare utilization is the ability for women “to go where they wish, when they wish,” and not only for them to be able to leave the home alone [32]. Some women in our study were allowed out of the home alone but were not given permission to go *where* they wanted. Considering access to mental health services is already difficult in India [12], understanding the nuanced dimensions of women’s freedom of movement and its link to building mentally healthy communities is critical.

Our study contributes key information on why family members restrict women’s movements and how some women, but not others, may leave the home to participate in PSSGs. For some women, their desire to participate in the support groups pushed them to find a way to leave the home, in some cases by lying to their family about where they were going. This secretive method of leaving the home is not sustainable or safe long term, however it underlines the importance of the groups in the women’s lives. In order to form communities that work together to become stronger, mental health must be discussed within households and across communities.

### Community mental health awareness

The above finding on women hiding their PSSG participation highlights the importance of community mental health knowledge, in order for families to understand the value of allowing women to learn about and strengthen their mental health. Therefore, our findings highlight the importance of pairing PSSGs with community mental health awareness and knowledge programs. Currently, Burans undertakes informal “corner meetings,” with the two-fold purpose of providing an opportunity for the community to build trust with Burans team members as well as to strengthen mental health knowledge in the community. The horizontal dialogue method used (as opposed to the more vertical and didactic approaches that are commonly used by health workers in India) led to a para-social interaction that supported learning, and provided opportunity for community members to develop new thought patterns and behaviours [44]. The next step to further increase mental health awareness could be to support dialogue between PSSGs and community members attending other programs.

### Women’s education and employment

Other key PSSG participation factors included the impact of education and employment, two important social determinants of health. Women with higher levels of education participated more actively within the groups, underlining that education provides an opportunity to build both social and communication skills. A study in Dehradun district, Uttarakhand, reported a strong correlation between reduced years of schooling and increased risk of depression [13], and the importance of education as a mental health determinant has been well described [45, 46]. The group participants with more education also reported greater opportunities for employment, thus proposing that one of the mediating pathways to protect from mental ill health is with increased education.

Our study found that employment outside the home gave women permission to leave the house. As such, there is a need to experiment with CMH program models that could, in a potentially more culturally acceptable manner, provide women with more freedom of movement, which, in turn, can facilitate access to community health resources including PSSGs. One solution would be to pair PSSGs with a microfinancing program, as one mental health program in India has done [27]. A combined intervention could allow women to collectively save money as a group, learn about banking and saving, and increase financial inclusion while gaining the benefits of the PSSG. We described this model to participants in the second round of FGDs, and women believed this addition could improve household income and women’s mental health while providing a more valid reason for women to leave the house.

## Implications

### Starting with communities: policy support for CMH competence

In the context of North Indian gender relations, it is important for policies and programs increasing women’s agency to be community-based. This is because individual-level programs for empowerment may be in conflict between “women’s new capacities for independent control and decision-making and the patriarchal cultural norms of their families and societies” [41]. This means that CMH Competence must be built across the entire community and supported by all community members. Initiatives focused solely on the individual “could result in negative or unintended outcomes for women” [41], for example, in cases where family members are not aware of the purpose of PSSGs.

The individual, household, and the community levels are intrinsically linked, and policy making in the area of women’s mental health needs to consider “evidence-based community, group and individual interventions targeting empowerment of women” [42]. PSSGs provide one such example, as they work at the community level, however, they impact gender relations and mental health stigma at the individual, household, and community levels. Although PSSGs were run by a local organization, NGOs themselves are rarely able to scale up services to provide wide coverage [47]. Therefore, funding for these community level initiatives must be prioritized at the state and national mental health budget levels.

### Gender relations: forefront of CMH programs

Community level programs addressing mental health must pay close attention to gender relations in order to optimize program participation. In the case of Northern India, it is clear that women’s autonomy is of paramount importance in enabling women to assemble collectively. In order to achieve this, Jejeebhoy and Sathar [28] suggest impacting women’s autonomy directly through various approaches including: “raising women’s gender consciousness, enabling women to mobilize and access community resources and public services, providing support for challenging traditional norms that underlie gender inequities, facilitating the acquisition of usable vocational and life skills, enhancing women’s access to and control over economic resources, and enabling women to establish and realize their rights.” The PSSGs set up by Burans begin to address each of these aforementioned areas, and therefore directly impact women’s autonomy, and their participation in community programs. Lastly, it is important for community mental health programs to incorporate other determinants of health including education, employment, and mental health awareness.

At the same time, the individual women themselves cannot be forgotten. For those who fought to access PSSGs, not only did their mental health improve and their confidence increase, but the women began sharing their mental health knowledge and referring community members for care (see our forthcoming publication on PSSG impact). As this individual-level knowledge sharing and service referral increases, community-level changes will increase mental health awareness, and build CMH competence over time. In turn, this may create a more supportive environment for women to attend PSSGs. However, this feedback loop emphasizes the importance of targeting women’s freedom of movement. If women cannot leave the home to participate in the support groups, this disables their opportunity to strengthen their own as well as the community’s mental health. As one group member shared:

*GM: “How will we share [what we have learned] with anyone when we don’t go out?”*

## Methodological considerations

This study summarizes its approach to rigour in four main areas: credibility, transferability, dependability, and confirmability [48]. One important way of increasing credibility is by building trust with participants. Because the study was embedded within the NGO and the support group’s CHW was present during FGDs, it provided a familiar environment to the women. PP and NG met each group two to three times in order to build trust to the point where the women opened up about what was preventing them from participating in the groups (this meant groups were visited even before the first FGD). Given the sensitive nature of some questions, approaching the women through the team members of a host organization they already knew well was deemed most appropriate. While there was a risk of social desirability bias when employees from the mental health program itself were involved in the FGDs, it is likely that the presence of community workers known to group participants supported more engaged and honest responses than had the lead author and an unfamiliar translator conducted the FGDs alone. However, it is important to highlight that this presence may have created gaps in the data related to aspects of the interventions on which the women did not feel comfortable commenting. Credibility was further strengthened by completing two rounds of FGDs, and using four types of triangulation: methodological triangulation (FGDs and KIIs), investigator triangulation (NG, KM, and PP), data triangulation (community members, psychiatrists, etc), and environmental triangulation (three separate sites). Investigator triangulation built consensus between researchers, while methodological, data, and environmental triangulation increased the corroboration of findings as the same themes appeared across different research sites, types of participants, and interview methods. For example, a KII with a male community leader revealed the same restriction on women’s freedom of movement that was declared in FGDs by the women themselves participating in PSSGs.

There was no non-response or refusal to participate by participants, although some women participated in the discussion more than others. We reiterated several times how participation was voluntary. Overall, the women participated enthusiastically. At the same time, this study has limitations due to its sampling, whereby, all women in FGDs were part of PSSGs; however, the perspectives of women outside of PSSGs were not included, meaning that the perspectives of women who could not participate in PSSG are not presented. This an important limitation for the study, as there may be other factors restricting PSSG participation, for example, types of mobility or accessibility barriers, of which we are unaware. However, accessing women with limited ability to leave their household and few connections to community programs may prove challenging. There is therefore a need for further study to be done, to engage women in the community who it may be difficult to reach, and examine the breadth of reasons for non-attendance to PSSGs. Lastly, the inclusion of male voices in the community would be an important area for further research to uncover the community’s attitudes around freedom of movement from within the household.

To be able to examine transferability to other contexts, this study used thick description in the form of notes on research context and process (oral and written records). Although some of the findings may be applicable to other community based mental health programs in South Asia, the context of Northern India is unique and therefore generalizations from this research should be examined alongside context descriptions. Lastly, dependability and confirmability was addressed by using reflexivity journals, audio recordings, and notes on research process. After each FGD and KII, NG and PP recorded an audio on their initial thoughts and any need for small improvements to the research process. This ensured that a paper trail detailed the research process, any changes made, and their justification (ex. better ways of phrasing questions). In terms of dependability, reflexivity allowed the researcher’s values and assumptions to be clearly stated, in order to better understand their impact on the interpretation of findings.

## Conclusions

Not only are mental health and gender inequality inseparable in the North Indian context, but women’s mental health cannot be addressed without first addressing underlying gender relations and norms that define socially acceptable activities for women outside the home. This study demonstrated that women’s freedom of movement was the most dominant factor restricting women’s access to community-based psychosocial support groups. However, our findings also spoke to the creative ways women showed agency despite the social obstacles that thwarted them, using strategies to leave home and assemble, which ultimately had beneficial consequences for them. In order for communities to grow and heal together while gaining new knowledge, women’s control over their movements is essential. Women’s autonomy as an essential component of CMH competence must be further studied, in order to enable community workers and the women they support to engage in effective interventions and to advocate for greater access to care and treatment in the community. To our knowledge, this is the first study to clearly document and analyze the connection between women’s freedom of movement and access to community mental health services in South Asia. Overall, policies prioritizing freedom of movement and gender equality may be more important for women’s mental health in this context than those focused on mental health service provision. To ensure all women can access mental health services, attention to the gender order that restricts women’s freedom of movement and their ability to access care is required.

## Additional files

Additional file 1: FGD Guide Round 1: focused on Community Mental Health Competence. (DOCX 18 kb)

Additional file 2: FGD Guide Round 2: focused on Freedom of Movement. (DOCX 14 kb)

Additional file 3: KII Guide. (DOCX 15 kb)

## Abbreviations

CHW: Community Health Worker
CMH: Community Mental Health
FGD: Focus Group Discussion
KII: Key Informant Interview
PPSD: Persons with Psychosocial Disability
PSSG: Psychosocial Support Group
