# Supplementary material for: Women’s freedom of movement and participation in psychosocial support groups: qualitative study in northern India
Source: BMC Public Health. 2019 Jun 10;19:725. doi: 10.1186/s12889-019-7019-3 (PMC6558745; doi:10.1186/s12889-019-7019-3)
Supplement: Supplementary file 1 — FGD Guide Round 1: focused on Community Mental Health Competence. (DOCX 18 kb) [file 12889_2019_7019_MOESM1_ESM.docx]

**FGD Guide Round 1: focused on Community Mental Health Competence**

Translated from Hindi

**Question 1**. Tell us your name and your favourite part about the support group.

**Question 2**. Let’s talk about your support group.

2a. What are some of the positive things that you have learned from joining this group?

2b. How has participating in this group changed your ideas on mental health?

2c. Has anything negative happened due to your participation in the group?

2d. When you first started coming to the groups, was anyone disapproving? (Probe: a neighbour? a family member?)

**Question 3**.

3a. Do you think more communities should have support groups like this one?

3b. Why or why not? If so, why are they important?

**Question 4.**

You said that (*eg. having support/learning about mental health*) was a great part about these groups. What has helped the group to be successful? Can you think of anything that would make them more successful?

**Question 5.** What advice would you give us in order to successfully form new support groups?

**Question 6**. Let’s talk more about your group.

6a. Whose support group is this? Do you feel that this is your group, or is it Burans’ group?

6b. How does that change your involvement in the group?

**Question 7**. Let’s imagine a new situation where your CHW does not lead the group anymore.

7a. What do you think would happen to your support group if ____(insert CHW name) did not come back to lead your group every week?

7b. What has happened in the past when the CHW can’t organize or attend the group?

**Question 8**

8a. How would your group function on its own? (without Project Burans’ support)

8b. What could make your group more likely to be able to continue on its own?

**Question 9**. Do you know what an SHG (self help group) is? (explain the concept for those who do not know it). Imagine if an SHG was combined with a support group: for the first hour, the group would be an SHG and the second hour, it would be a support group.

9a. What do you think about combining a support group with an SHG?

9b. What would be the positive and negative aspects of combining an SHG with a support group?

**Question 10**

These are all important questions when we think about the long term success and initiatives like support groups that are based on collective action/collaborative action in communitites.

*One great example in your community of collective action is a group in Sehaspur (neighbouring town) called the DPG (disabled persons group). They advocate for the rights of mentally and physically disabled people, helping them to get money from the government to help in times of disability. They started the group themselves, with 3 members. Then, with the help of an NGO (EHA), they got trainings and help, and grew bigger. Now they have over 300 members, and have become their own organization. They go into other villages and start small DPG support groups, with their members.*

*This is a great of example of a group that has formed on their own, with some help from an organization; however, the ownership of the group is their own. They are not dependent on an NGO to help them every week.*

10a. What do you think about this DPG?

10b. What do you think would be the positive and negative aspects of your support group taking action to do something like this?

10c. To grow the support groups in other communities would require energy and time. Does anyone here think they would be willing to work towards this? Please raise your hands.
