# Supplementary material for: Women’s freedom of movement and participation in psychosocial support groups: qualitative study in northern India
Source: BMC Public Health. 2019 Jun 10;19:725. doi: 10.1186/s12889-019-7019-3 (PMC6558745; doi:10.1186/s12889-019-7019-3)
Supplement: Supplementary file 2 — FGD Guide Round 2: focused on Freedom of Movement. (DOCX 14 kb) [file 12889_2019_7019_MOESM2_ESM.docx]

**FGD Guide Round 2: focused on Freedom of Movement**

Translated from Hindi

**Question 1:** We have come here to talk about some of the results we found after discussions we had with you and other support groups. The first thing we will discuss is about leaving your home. In the first round of discussions with several support groups, it came out that women find it difficult to go out of the home. Do you also have problems or difficulties in coming out of your home?

**Question 2**: Why do you have difficulty?

**Question 3:** What do your families think about your coming to this support group?

**Question 4:** When you first started coming to the group, were there any challenges? Did anyone stop you from coming?

**Question 5:** Imagine there is a woman who is not allowed to leave home to go to the group. What can we tell the woman to make her come to the group meeting? How can we help her to come?

**Question 6**: Do the people of your community know that you are part of a support group? What do they think about the support group?

**Question 7**:

7a. Do you think you are able to speak openly in your group?

7b. For those who say yes: do you think participating in this group has impacted your ability to speak openly with other people *outside* the group (eg. family members)?

**Question 8:** How do you feel about the information related to mental health, which you received by joining this group?

**Question 9**: Are you able to share mental health information with others after joining the group?

**Question 10:** We will now talk about creating independence for the support groups. Imagine Burans is not there to run the groups sometime in the future. Would your group be able to continue on its own or would it likely stop?
