# Supplementary material for: Women’s freedom of movement and participation in psychosocial support groups: qualitative study in northern India
Source: BMC Public Health. 2019 Jun 10;19:725. doi: 10.1186/s12889-019-7019-3 (PMC6558745; doi:10.1186/s12889-019-7019-3)
Supplement: Supplementary file 3 — KII Guide. (DOCX 15 kb) [file 12889_2019_7019_MOESM3_ESM.docx]

**KII Guide**

**Question 1:** Tell me a bit about yourself, where you work and your position.

What has been your experience working with women with depression and anxiety?

**Question 2:** I notice that Burans has been teaching communities about mental health. What differences do you notice in the patients that come to you, as a result of Burans’ work?

For example, have their notions/knowledge of mental health changed? How?

**Question 3:** Let’s talk about women’s independence, for example, their ability to leave the home to come to hospital or travel, or leaving home to go to community-lead support groups.

3a. How independent is the average woman that visits you?

3b. How far from her house is she allowed to go alone?

**Question 4:**

4a. What factors in the community *enable* women to have more independent action?

4b. What factors in the community *restrict* women’s independent action?

4c. Have you seen any changes over time, and what do you think has contributed to this?

**Question 5:**

5a. Which member of the family is most likely to restrict a woman’s movements? How could this be combated to help ensure more support groups can be made?

5b. Others have suggested that engaging the family and talking to them about what the women gain from the support groups would help. What do you think? Who in the family would be most important to convince?

5c. Women say they are monitored and watched by the rest of the community, which may prevent them from going to a group. What could help the community to see the women in PSSGs differently?

**Question 6:**

I also want to get a better understanding of local women’s ideas of ownership in their own lives.

5a. What kind of things do they feel ownership over?

5b. Do they feel ownership over their health?

**Question 7:** Let’s talk about collective action for mental health.

6a. What does community/collective action mean to you? What types of actions does it include?

6b. What do you believe are the most urgent priorities for the community’s mental health and how could they take action collectively to achieve this?

**Question 8:** I’m interested in understanding how community actions can become more sustainable over time. Right now, I notice a dependency on the NGO (Burans), which is natural.

What do you think would help reduce this, and eventually allow women to run support groups on their own, even create new groups in other communities?
